# Japanese medical learners’ achievement emotions: Accounting for culture in translating Western medical educational theories and instruments into an asian context

**DOI:** 10.1007/s10459-021-10048-9

**Published:** 2021-05-12

**Authors:** Osamu Nomura, Jeffrey Wiseman, Momoka Sunohara, Haruko Akatsu, Susanne P. Lajoie

**Affiliations:** 1 Department of Emergency and Disaster Medicine, Hirosaki University, 5 Zaifu-cho, Hirosaki, Japan; 2 Department of Educational & Counselling Psychology, McGill University, Montreal, Canada; 3 Institute of Health Science Education, McGill University, Montreal, Canada; 4 Department of Psychology, Concordia University, Montreal, Canada; 5 Medical Education Office, International University of Health and Welfare, Narita, Japan

**Keywords:** Control-value theory, Clinical reasoning, Emotions, Measurement, Japanese

## Abstract

Medical learners’ achievement emotions during educational activities have remained unexamined in Asian cultural contexts. The Medical Emotion Scale (MES) was previously developed to assess achievement emotions experienced by North American medical learners during learning activities. The goal of this study was to create and validate a Japanese version of the Medical Emotion Scale (J-MES). We translated the MES into Japanese and conducted two initial validation studies of the J-MES. In the first pilot study, we asked five, native-Japanese, second-year medical students to assess their emotions with the J-MES during a computer-based clinical reasoning activity. Each participant was then interviewed to assess the clarity and suitability of the items. In a second, larger study, 41 Japanese medical students were recruited to assess the psychometric properties of the J-MES. We also conducted individual, semi-structured interviews with ten of these participants to explore potential cultural features in the achievement emotions of Japanese students. The first pilot study demonstrated that the J-MES descriptions were clear, and that the scale captured an appropriate range of emotions. The second study revealed that the J-MES scale’s profiles and internal structure were largely consistent with control-value theory. The achievement emotions of pride, compassion, and surprise in the J-MES were found to be susceptible to cultural differences between North American and Japanese contexts. Our findings clearly demonstrated the scoring capacity, generalizability, and extrapolability of the J-MES.

**Supplementary Information:**

The online version contains supplementary material available at 10.1007/s10459-021-10048-9.

## Introduction

### Role of emotions and its definitions in medical education

Clinical experience in healthcare settings is fraught with emotional situations, both positive and negative, such as joy at achieving self-development, pride in one’s professionalism, grief and guilt over the death of a patient, fear of malpractice suits, and hopelessness when caring for critically ill patients (McConnell & Eva, 2012; Pitkälä & Mäntyranta, 2004). Similarly, clinical medical education involves situations potentially charged with emotion. Most of the literature on the emotions in health profession education focuses on negative emotions which are believed to reduce physicians’ wellness (Croskerry et al., 2008; Nomura et al., 2016). Some degree of anxiety may enhance learning in trainees; however, excessive anxiety inhibits effective learning (Kasman et al., 2003). Positive emotions in medical students can be a predictor of their achievement on tests (Artino et al., 2010). Whether positive or negative, medical trainees experience a range of intense emotions that can influence their ability to learn, teach, and apply their skills to new settings.

Emotion is only beginning to be studied in medical education since traditionally it has been considered to be “non-cognitive” and therefore irrelevant (Artino et al., 2012). Shapiro (2011) has cited physicians’ alexithymia, the “difficulties in recognizing, processing, and regulating emotions” (Taylor et al., 1999) and suggested that doctors, “work with emotions,” i.e., engage in identifying emotions, evaluate their appropriateness and usefulness for achieving patient-centered goals, and regulate them properly. In medical education research, few experimental studies have examined medical trainees’ affective constructs, such as “achievement emotions,” the feelings related to achievement activities or achievement outcomes in learners (Artino et al., 2011; Jarrell & Lajoie, 2017; Pekrun & Perry, 2014). In addition, education concerning emotions is not offered in most medical school curricula (de Vries-Erich et al., 2016; Satterfield & Hughes, 2007). However, there are recent calls for more attention to be paid to research on emotions and for including education on emotions in the medical school curriculum (McConnell & Eva, 2012).

There are several key emotion constructs that are pertinent in a medical education context. There are two distinct types of affective states, moods and emotions, that are distinguished from each other by their intensity and duration (Rosenberg, 1998). Moods tend to be longer, broader, and lack a specific cause (e.g., feeling stressed) whereas emotions tend to be shorter, more intense, and have a specific cause (e.g., feeling stressed during this morning’s clinic when confronted with multiple unexpected patients) (Forgas, 2004). Artino et al., (2012) define emotion in the medical education context as “a psycho-physiological change that is short-lived, intense and specific to a personally meaningful stimulus.” Furthermore, Pekrun (2006) has defined achievement emotions as, “emotions tied directly to achievement activities or outcomes.” In clinical contexts achievement emotions would be tied to activities like solving clinical problems, performing surgical procedures, and communicating difficult news or to outcomes such as medical errors and clinical performance evaluations.

### Control-value theory of achievement emotions

**Fig. 1:**
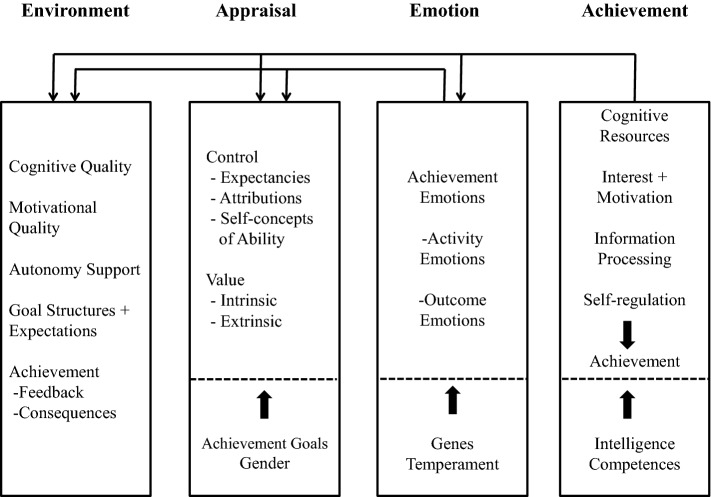
Control-value theory

Pekrun’s control-value theory (Fig. 1; Pekrun & Perry, 2014) postulates that learners’ achievement emotions will influence the degree to which they learn to do a given task. Learners’ achievement emotions are modulated by learners’ prior perceptions of the degree to which they have control over and value a given task. Learners’ control and value perceptions are in turn influenced by environmental factors perceived when learning occurs, thus linking achievement experiences, achievement emotions, cognitive appraisals (i.e., control and value) and environmental factors together in a self-regulating cycle. In control-value theory, achievement emotions are classified into three dimensions: valence (positive versus negative), arousal (activating versus deactivating), and object-focus (activity-related versus outcome-related). Pekrun et al. developed a self-report scale based on control-value theory called the Achievement Emotions Questionnaire (AEQ; Pekrun et al., 2002, 2005). The AEQ is a multidimensional assessment tool originally designed to evaluate college students’ achievement emotions and consists of items assessing the classroom learning-related enjoyment, pride, anger, anxiety, shame, hopelessness, and boredom. Artino et al., (2010, 2011), using the AEQ as their measurement method, showed that positive course-related emotions (e.g., enjoyment or happiness) positively predicted medical students’ academic performance on the national board shelf examination while negative emotions (e.g., boredom or anxiety) negatively predicted academic performance on the same test. Other studies measuring medical students’ achievement emotions during a specific task, such as computer-based clinical reasoning activities, have shown similar results (Harley et al., 2019; Jarrell et al., 2016, 2017; Naismith & Lajoie, 2018). These studies pioneered the use of AEQ based on control-value theory for research on emotions in the medical education context; however, the AEQ was originally developed to assess students’ emotions in traditional classroom settings and therefore the validity of the AEQ for measuring medical trainees’ emotions on clinical tasks remains unexamined.

Duffy et al., (2018) developed a medical education achievement emotion scale by selecting elements of the AEQ that were more relevant to medical educational contexts. While the AEQ included three different sentences to measure a single emotion in multiple time points (before during and after), the new scale utilizes single-word adjective to describe the emotions at these time points. The resultant Medical Emotion Scale (MES; Supplementary material) is a self-report scale containing 20 items designed to measure different types of achievement emotions in medical education contexts with a five-point Likert scale to estimate the perceived intensity of each emotion. Each emotion is characterized by valence (positive or negative) and arousal level (activating versus deactivating) to create four categories of medical emotions: (1) positive activating (PA), (2) positive deactivating (PD), (3) negative activating (NA), and (4) negative deactivating (ND) emotions (Supplementary material). Like the AEQ, the MES aims to measure emotional states at multiple time points (before, during, and after a given task). The AEQ included three sections for measuring students' emotions in classroom, learning, and test environments respectively; however, the MES focused on measuring the leaners' emotions in the authentic problem-based learning environment.

Duffy et al., (2018) conducted validation studies of the MES with North American subjects across multiple, authentic, live and technology-based medical educational environments and showed that the internal reliabilities(α) for each sub-scale of the MES were high: PA (0.91), PD (0.69), NA (0.92), ND emotions (0.88). The studies also confirmed that the emotions measured using the scale aligned with the conceptual understanding of the construct of emotions as described by control-value theory.

### Creating a Validation Argument for the Japanese Medical Emotion Scale

Culture influences the experience and expression of emotions in the school setting (DeCuir-Gunby & Williams-Johnson, 2014). Asian students are more likely than Western student to experience feelings of shame and dishonor (both for themselves and their families) with unfavorable academic achievement outcomes (Zeidner, 2014). Similarly, in medical educational settings, culture influences the emotions experienced by medical trainees and professionals (Artino & Naismith, 2015; Helmich et al., 2017). However, the focus of the majority of studies in this area outside the West has been limited to negative emotions (Cheung & Au, 2011; Yusoff, Abdul Rahim, et al., 2013; Yusoff, Esa, et al., 2013). The MES (Duffy et al., 2018) as described above was created for use in the Canadian medical education context and has neither been translated into other languages nor validated for use in other medical educational cultures. Therefore, in order to examine Japanese medical students' achievement emotions, the MES need translation into Japanese to create a Japanese version (J-MES) and validation of the J-MES in Japanese medical educational settings.

**Fig. 2:**
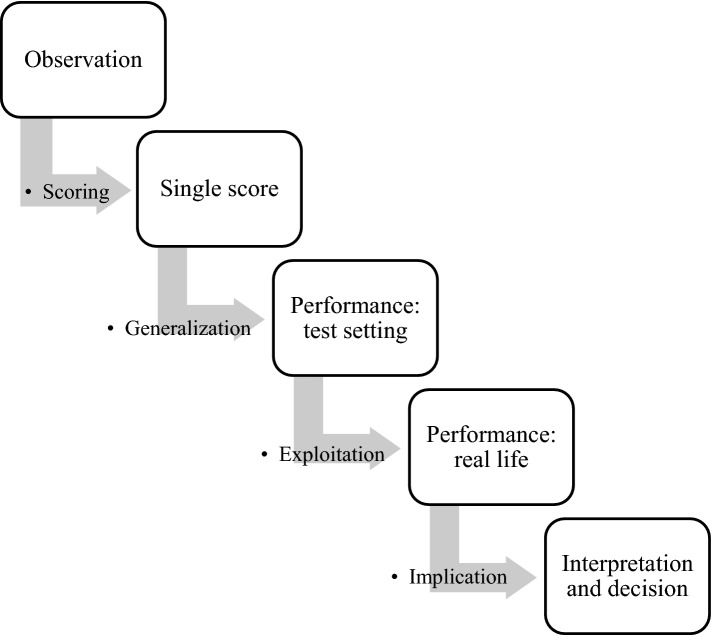
Kane’s Key elements in the validity argument

According to Kane (2013) validation of any measurement method requires gathering evidence to examine the four key inferences one must make in order to contribute to an argument that measurement results are or are not believable for a given context, use and decision: (1) scoring of a single observation (Scoring); (2) using the primary observation score data to generate a picture of overall test performance (Generalization); (3) inferring real-life performance from test performance (Extrapolation); and (4) interpreting this information and making a decision (Implication) (Fig. 2). Kane’s framework makes it possible to see where previous validation studies fit into an overall validity argument as a chain of accumulating evidence, thus highlighting the gaps that future studies like those of the J-MES can address. Kane’s framework also permits incorporation of not only quantitative but also qualitative evidence from analyses of the cross-cultural perspectives of Canadian and Japanese medical trainees (Cook et al., 2015) to create a validation argument for the J-MES.

## Research questions

The present study addressed the following research questions (RQs):

1. How do Japanese medical students interpret and respond to the J-MES?
2. Does the J-MES represent an adequate range of emotions that medical students may experience in a technology-rich learning environment?
3. Are there any cross-cultural features in the perception and manifestation of emotions in Japanese medical students?
4. Do emotions measured by the scale reflect theoretical constructs, including perceived control and value, performance, and post-task self-efficacy?

RQs 1 and 2 were addressed in the “Pilot phase” of this study using a qualitative methodology to assess the J-MES scoring and generalization inferences, respectively. In the “Finalization phase” of this study, RQ 3 was answered using a qualitative approach to assess the J-MES extrapolation inference, and RQ 4 was addressed using quantitative methods in order to examine the implication inferences of the J-MES. (Fig. 2). Table 1 shows the structure of this study.

**Table 1:** Methods: Validation of the J-MES

Research question	Kane inference type	Types of data collected	When in relation to the BioWorld activity
Pilot Phase			
(1) How do Japanese medical students interpret and respond to the J-MES?	Scoring	Semi-structured interview data	After the activity
(2) Does the J-MES represent an adequate range of emotions that medical students may experience in a technology-driven learning environment?	Generalization	J-Messemi-structured interview data	Before, during after the activity After the activity
Finalization Phase			
(3) Are there any cross-cultural features in the perception and manifestation of emotions in Japanese medical students?	Extrapolation	Semi-structured interview data	After the activity
(4) Do emotions measured by the scale reflect theoretical constructs, including perceived control and value, performance, and post-task self-efficacy?	Implication	Prior knowledge Control Value J-MES Self-efficacy Performance of BioWorld	Before the activity Before the activity Before the activity Before, during after the activity After the activity Whole process in the activity

## Methods

### Development of the Japanese version of the Medical Emotion Scale

**Fig. 3:**
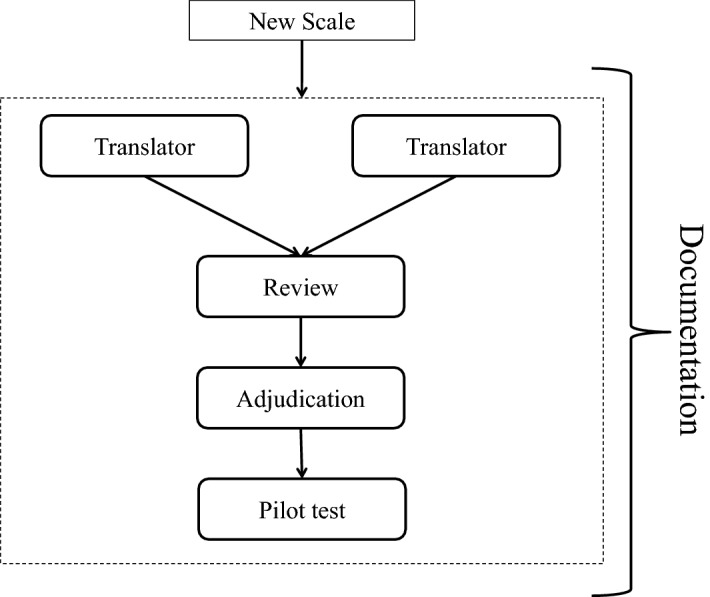
Translation, Review, Adjudication, Pretest, Documentation team model

The J-MES was developed by translating the MES into Japanese using the Translation, Review, Adjudication, Pretest, Documentation (TRAPD) team translation model (Survey Research Center, 2016), which follows cross-cultural survey guidelines to ensure proper translation and cross-cultural and linguistic equivalences between the English and Japanese survey versions. We formed a bilingual translation committee consisting of translators, a translation reviewer, and an adjudicator (Fig. 3). Two translators who are currently graduate students pursuing linguistics and second language education, respectively, translated the entire MES into Japanese individually, and then reviewed each other’s version. Then, one reviewer, a graduate student in cultural psychology, examined all the items and identified those with potential problems from a cross-cultural perspective. The committee convened two, three-hour meetings to discuss all the items and any issues and to improve the accuracy of the translation. The principal investigator adjudicated the final version of the J-MES with the agreement of the translators and reviewer (Supplementary material). The TRAPD team model established linguistic equivalency between the J-MES and its English original, enabling us to discuss cross-cultural perspectives on Japanese medical students’ emotions as measured by the scale.

### Participants and learning environment

The participants were native Japanese-speaking, second-year medical students attending a medical school in Japan where the North American-style preclinical medical education curriculum is taught in English. Students were recruited using a flyer explaining the subject and purpose of the study and the details of their participation. Students were informed that participation was voluntary and that their anonymized data would be used for research purposes and would not influence their academic standing. Written informed consent was obtained from the participants prior to data collection and participants were allowed to withdraw at any time. All the participants received a 2000 JPN yen Amazon gift certificate upon completing their participation. The study was reviewed and approved by the Research Ethics Board at a North American University and the Research Ethics Committee at a Japanese Medical School.

We utilized BioWorld (Lajoie, 2009), a computer-based learning environment that helps medical learners develop diagnostic reasoning skills through solving standardized medical cases with undifferentiated presentations. For instance, participants were presented with a BioWorld “patient” complaining of frequent urination and thirst and had to work their way through selecting and interpreting pertinent details from the “patient’s” history, physical examination findings, and simulated laboratory tests to arrive at a diagnosis of diabetes mellitus. BioWorld is useful for examining emotions in preclinical medical students as it presents identical medical case scenarios appropriate to the preclinical student level. In addition, BioWorld is the same learning environment used to develop the English Canadian version of the original MES and thus affords a comparable medical educational context for the J-MES validation study. The language used in the BioWorld activity in this study was English, which is the language of instruction at this Japanese university.

### Pilot phase study of the Japanese version of the Medical Emotion Scale

The goal of the pilot study was to collect evidence of the validity of scoring and generalization interferences in Kane’s framework by qualitatively examining how Japanese medical students interpreted the meaning of the scale items (RQ 1) and whether the J-MES represented an adequate range of emotions (RQ 2) (Table 1).

Five, second-year medical students were randomly chosen to take part in piloting the J-MES. The group size was based on previous validation studies (Duffy et al., 2018; Oishi et al., 2017). The five participants were first given the J-MES “before” being given the BioWorld task, then responded to the “during task” J-MES questionnaire immediately after the activity, and finally completed the “after task” J-MES questionnaire following their completion of the BioWorld task. Afterwards, the participants took part in a semi-structured interview (approximately 15 min) and were asked to explain their perceptions and interpretations of the scale to determine whether it captured a representative range of emotions, whether it was interpreted in a manner consistent with the researchers' assumptions, and whether it was suitably formatted for responses. The interview questions and protocol of the original MES study were translated into Japanese using the TRAPD method (Supplementary material). The interviews were audio-recorded, transcribed, and member-checked.

#### The Japanese version of Medical Emotion Scale

The Japanese Version of the Medical Emotion Scale (J-MES; Supplementary material) consists of 20 items containing adjectives describing discrete emotions for assessment using a five-point Likert scale (1 = not at all to 5 = very strongly). The items are categorized into four subscales according to the valence (pleasant/unpleasant) and activation (level of physiological arousal) of the emotions: (a) positively activating (e.g., joyful, curious); (b) positively deactivating (e.g., relaxed, relieved); (c) negatively activating (e.g., anxious, frustrated), or (d) negatively deactivating (e.g., hopeless, disappointed). In addition, the “during” questionnaire was administered immediately after the task to prevent interference with the task because in the original MES study, retrospective accounts of emotions were found to be highly aligned with emotions measured concurrently with activities lasting approximately 1 h.

#### Analyses

The positive and negative emotions assessed by the J-MES across the three time periods were described statistically (i.e., mean and standard deviation). The qualitative data were collected via individual, semi-structured interviews held after the BioWorld activities. We conducted content analysis of the interview data using a modified coding scheme of sensibility assessment (O'Brien et al., 2013), a question-feature-based coding system assessing the participants’ perception of a scale’s clarity, the items’ suitability for representing the participants’ emotions, the format’s usability, and additional, emerging themes (Table 2; Supplementary material). We also aimed to explore cross-cultural differences in emotions between Japanese and North American medical students. Two researchers independently coded the qualitative data and compared their results after coding the first two participants’ data to align their results. They again independently coded the last three participants’ data using the prescribed coding method. Finally, the two researchers analyzed the coded data together and completed the qualitative content analysis. Coding differences were resolved by discussion between the two researchers.

**Table 2:** Qualitative interview results by coding scheme

Coding schemes	Sample quotations
Suitability	*[These items] cover most of the emotions that I felt.* *There are no emotions that need to be added.*
Clarity	*I was able to understand the meaning of most items.*
	*None of the items was difficult to understand.*
Usability	*This questionnaire was easy to answer.*
	*The option buttons were convenient to use.* *The use of color in the questionnaire was excellent.*
Additional emerging themes (Items requiring further investigation)	Compassion:* "Compassion" doesn't make sense. Compassion for what? I don’t feel compassion when solving problems. "Compassionate" might make sense when working in a team but doesn’t in this situation.*
	Proud: *I don’t feel proud while studying.* *We don’t feel pride towards ourselves.* *Few people would feel proud while solving a problem.*

### The finalization phase validation studies of the J-MES

After the pilot study, the finalization phase was implemented to examine evidence of the validity of Kane’s extrapolation and implication inferences (RQ 3 & 4). (Table 1). Participants not allocated to the first pilot study participated in the finalization phase of J-MES validation.

**Participants** Forty-one, second-year medical students joined the final phase of J-MES validation. The sample size was based on previous studies using MES (Duffy et al., 2018; Lajoie et al., 2018). To add qualitative evidence to this cross-cultural version of the MES, the students were invited to take part in an individual qualitative interview through convenience sampling. Ten students agreed to participate in the semi-structured interview.

**Demographics and prior knowledge** Demographic data, such as age and gender, were collected, and participants reported their prior knowledge of diagnostic reasoning using a 7-point Likert scale (1 = no knowledge at all to 7 = a great deal of knowledge).

**Control** Academic control (Perry et al., 2001) was assessed using the five items of the Japanese version of the Academic Control Scale to measure their cognitive appraisal of control-toward-performance (Ikeda, 2015). (*α* = 0.75) (Supplementary material).

**Value** The perceived value of the task (Miyabe et al., 2016) was assessed using the six items on the Japanese version of the Motivated Strategies for Learning Questionnaire (*α* = 0.87) (see Supplementary material).

**Emotions** As described above, learners’ emotions were assessed with the J-MES administered before, during, and after the BioWorld activity.

**Self-efficacy** Post-task self-efficacy was also assessed after task completion using a single item adapted from Bandura’s scale (Bandura, 2006) to measure participants’ confidence when performing a similar task in the future. A 100-point Likert scale (0 = no confidence to 100 = complete confidence) with 10-point intervals was used to measure confidence levels. The original English scale was translated into Japanese using the same procedure as in the translation of the MES (Supplementary material).

**Performance** Performance on the BioWorld environment diagnostic reasoning skills test was used to assess the correctness of the participants’ diagnosis (Supplementary material). The results were obtained via log-files in the BioWorld software (Lajoie, 2009).

**Cross-cultural differences in emotions **Individual, semi-structured interviews (approximately 15 min for each participant, done after completion of the BioWorld activity were conducted to collect qualitative evidence for the cross-cultural content of the J-MES (Supplementary material). In these interviews, we focused on specific items that were susceptible to cross-cultural differences based on the results of the pilot study and previous studies (Imada & Ellsworth, 2011; Leu et al., 2011). The interviews were audio-recorded, transcribed, and member-checked.

### Analysis

**Correlation analysis** We performed a bivariate correlation analysis of prior knowledge, perceived control, value, and emotions to determine their relationship with each other.

**Regression analyses** Two logistic regression analyses were done to examine:

1. The relationship between performance on BioWorld, which was treated as a binominal variable (i.e., whether each participant’s diagnosis was correct or incorrect) and emotions measured by the J-MES. Positive activating, negative deactivating, and neutral emotions experienced before and during the task were treated as independent variables based on a previous study (Lajoie et al., 2018).
2. The relationship between self-efficacy which was treated as a continuous variable and emotions measured by the J-MES. Positive activating, positive deactivating, and negative deactivating emotions before and during the activity were treated as independent variables. All data were analyzed using SPSS, version 23 (Armonk, NY: IBM Corp).

**Qualitative Analysis** Content analysis of the interview data with the ten subjects for cross-cultural differences in emotions was conducted in the same way as in the first pilot study using a modified coding scheme of sensibility assessment (O'Brien et al., 2013; Supplementary material). See above.

## Results

### Pilot study results

**RQ 1** Five, second-year Japanese medical students (*n* = 3 female), with a mean age of 22.4 (*SD* = 4.8), participated in the pilot test. In the qualitative interviews (Supplementary material), the participants responded that the descriptions of the J-MES items were generally clear and the J-MES format was also user-friendly (Table 2). Some participants, however, commented that compassion was irrelevant to individual activities in a computer-based environment (e.g., BioWorld) but was more relevant to group learning. The participants also indicated that they "did not feel proud of themselves" in the BioWorld learning context.

**RQ 2** All five interview participants responded that the range of items adequately represented the emotions felt during the BioWorld diagnostic reasoning task (Table 2). In terms of quantitative data, the five, most intense emotions experienced in the full range of BioWorld activities are show in online supplementary material. Curiosity was the most intense positive emotion reported before (*M* = 3.8, *SD* = 1.2) and during (*M* = 2.8, *SD* = 0.7) the task. In the after-task phase, the most highly rated positive emotion was relaxation (*M* = 3.4, *SD* = 1.0). The most frequently reported negative emotion before the task was anxiety (*M* = 3.0, *SD* = 1.4) while confusion was most frequently reported during the task (*M* = 3.6, *SD* = 1.0). Finally, shame (*M* = 2.6, *SD* = 1.6) was the most frequently reported negative emotion after completion of the task.

Based on the results of the pilot study, the TRAPD team reached the consensus that the J-MES was translated accurately and could be used for statistical validation. In addition, the team determined that the items of “compassion” and “pride” required further qualitative analysis, which was subsequently done as part of the finalization phase of J-MES validation.

### Results of the finalization phase of J-MES validation

**RQ 3** We analyzed the qualitative data from ten participants’ individual interviews asking about their perceptions of the J-MES items of pride, surprise, and compassion (Supplementary material), which required further examination based on the results of the pilot study and previous studies (Table 2). Regarding each item, participants responded that “pride” was potentially problematic due to cross-cultural differences between Japan and Western countries. They reported that expressing one’s pride about oneself to others is discouraged in Japanese culture and that in general it is less common for Japanese to express this emotion in daily life. Although participants commented that they were able to understand the meaning of the items pertaining to “surprise”; however, they noted that surprise is a neutral item and its valence can be positive or negative depending on the situation. For example, students can feel positive surprise on discovering a topic to be more interesting than expected while they might feel negative surprise if their performance or perceptions of the learning environment are worse than expected. They also responded that “compassion” was difficult to understand and was unsuitable for assessment in a computer-based learning environment. They suggested that it might be more appropriate to assess compassion in a learning environment where students collaborate with others, such as group-work, simulations and real interactions with patients. The qualitative interview data was summarized in Table 3.

**Table 3:** Content analysis results for pride, surprise, and compassion items

	Sample Quotations		
Coding Schemes	Pride	Surprise	Compassion
Clarity	*I could understand "pride", but some people may feel uneasy with it.* *I was confused a bit about the meaning of “pride”. “Pride” might be difficult to understand for “typical” Japanese people.*	*The meaning of surprise was clear.* *I could understand “surprise”.*	*“Compassion” was the most difficult item to understand. I am not sure in what situation I could feel compassion.*
Suitability	*I was not sure what I could feel proud of. Could I feel proud of myself, for what I completed in the activity or the fact that I had this opportunity?* *“Satisfied” might make more sense.*	*Surprise is a neutral emotion and can be either positive or negative.* *The meaning of surprise changes depending on my performance. *	*“Compassion” would make sense if I had partners to work with.* *I did not get what compassion is for. It may be empathy for patients, but the patient this time was just a simulation.* *It was difficult to feel compassion as I was unable to see the patient's face. So, we tended to focus on problem-solving rather than responding to patients’ worries.*
Emerging theme	*While I lived abroad in my childhood, school teachers often said to me “I’m so proud of you”. So, I naturally felt “proud” of myself at that time, but Japanese teachers rarely say that. They say, “you are great”, but they never say “I am proud of you”. So, ‘pride’ might not be clear to some Japanese people. Pride is natural to me, but some Japanese students are not used to comments like, “I’m proud of you”.* *We Japanese don’t use the word "pride" so much. We don't say to others, "I'm proud of you" or "I'm proud of myself" compared to people in Western countries.* *Even if I believe that my kid is the smartest and feel proud of my kid in my heart, I would say to others, “My child is not as good as your child”* *When I am talking with close friends, I can say, “I’m proud of myself”. But, I do not express the emotion if the relationship is not so intimate. We should not speak of being proud because this comment may offend other.* *Showing pride may indicate that I am aiming to display superiority to the person I am speaking to.*	*I feel positive surprise when I learn something new that piques my curiosity.* *I would feel negative surprise if my assumptions were completely proved false. For example, on tests, if my answer to a question was incorrect when I was confident I was right, my surprise would be negative.* *It can be both (positive or negative). I would be positively surprised if I realized that I was doing better than expected, which would stimulate my intellectual curiosity. I would feel negative surprise if my learning environment, such as the educational software or program, was not as good as expected. So, expectations may influence whether surprise is positive or negative.*	*I would feel compassion in a learning setting involving group-work or simulation of a medical interview where we collaborated with others.* *I would be compassionate when working with patients or when imagining the presence of real patients.*

**RQ 4** Forty-one, second-year Japanese medical students (*n* = 19 females) with a mean age of 22.3 (*SD* = 3.8) participated in the finalization phase of this study.

The internal reliability level (*α*) of the J-MES measured before, during, and after the task was .66, .76, and .75, respectively, all of which were higher than the reference value of .6. Table 4 shows the descriptive statistics for perceived control and value, prior knowledge, emotions, self-efficacy, and performance across the three time points (before, during, and after the activity). The bivariate Pearson’s correlation analysis results were shown in Table 5. We found that control positively correlated with PA emotions experienced by participants before and after the BioWorld clinical reasoning activity while the value negatively correlated with NA emotions after the clinical reasoning activity. There was a positive correlation between prior knowledge and PA emotions before the activity. In terms of the links between emotions across the time points, PA emotions before the BioWorld task positively correlated with PA and neutral emotions during the performance of the task and with PA emotions after completion of the task. PD emotions before the task positively correlated with PA emotions both during and after the task. ND emotions before the task negatively correlated with positive activating and neutral emotions during the task and with PD emotions after the task. There was a positive correlation between neutral emotion before the activity and neutral emotion during the task. In terms of emotions experienced during the task, PA emotions positively correlated with PA, PD, and neutral emotions after completion of the task. PD emotions during the task positively correlated with PA and PD emotions, and negatively correlated with NA and ND emotions, after the task. Both NA and ND emotions negatively correlated with PD emotions and positively correlated with NA and ND emotions after the task. There was a positive correlation between neutral emotion during the task and PA and neutral emotion after the activity. Post-task self-efficacy positively correlated with PA and PD emotions both during and after the task and negatively correlated with NA and ND emotions both during and after the task.

**Table 4:** Descriptive statistics of variables across time

	Appraisals	Before	During	After	Performance
	*M* (*SD*)	*M* (*SD*)	*M* (*SD*)	*M* (*SD*)	*M* (*SD*) or *n* (%)
Prior knowledge	3.2 (1.3)	−	−	−	−
Control	4.1 (0.6)	−	−	−	−
Value	5.6 (0.7)	−	−	−	−
Pos. act. emotions	−	2.9 (0.9)	2.7 (0.9)	2.7 (1.0)	−
Pos. deact. emotions	−	3.0 (0.9)	2.9 (0.9)	3.2 (1.1)	−
Neg. act. motions	−	1.7 (0.5)	2.0 (0.7)	1.7 (0.7)	−
Neg. deact. emotions	−	1.4 (0.5)	1.8 (0.8)	1.7 (0.9)	−
Neutral emotion	−	2.4 (1.4)	2.4 (1.2)	2.1 (1.2)	−
Self-efficacy	−	−	−	−	55.4 (22.5)
Performance	−	−	−	−	21 (51.2)

pos. act. emotions = positive activating emotions; pos. deact. emotions = positive deactivating emotions; neg. act. emotions = negative activating emotions; neg. deact. emotions = negative deactivating emotions

**Table 5:** Correlation between control, value, prior knowledge, emotions, self-efficacy

Variables	1	2	3	4	5	6	7	8	9	10	11	12	13	14	15	16	17	18
1. Control																		
2. Value	.39^*^																	
3. Prior knowledge	.24	.23																
4. Pos. act. emots. (bef)	.33^*^	.27	.36^*^															
5. Pos. deact. emots. (bef)	.12	.25	.22	.44^**^														
6. Neg. act. emots. (bef)	−.03	−.19	−.09	−.10	−.33^*^													
7. Neg. deact. emots. (bef)	−.15	−.13	−.03	−.47^**^	−.21	.46^**^												
8. Neutral emotion (before)	.21	.28	.08	.45^**^	.03	.32^*^	−.16											
9. Pos. act. emots. (dur)	.27	.03	.06	.59^**^	.34^*^	−.13	−.35^*^	.11										
10. Pos. deact. emots. (dur)	.25	.17	−.19	.08	.20	−.03	−.11	.01	.45^**^									
11. Neg. act. emots. (dur)	−.23	−.08	.05	.28	.12	.29	.12	.22	−.06	−.47^**^								
12. Neg. deact. emots. (dur)	−.18	−.17	.03	.10	−.02	.29	.26	.09	−.26	−.47^**^	.82^**^							
13. Neutral emotion (dur)	.20	−.03	−.04	.47^**^	.17	−.06	.34^*^	.35^*^	.54^**^	.04	.37^*^	.23						
14. Pos. act. emots. (aft)	.36^*^	.14	.06	.58^**^	.34^*^	−.25	−.28	.13	.87^**^	.43^**^	−.04	−.24	.57^**^					
15. Pos. deact. emots. (aft)	.26	.30	−.11	.22	.19	−.15	−.32^*^	−.00^†^	.56^**^	.67^**^	−.40^*^	−.57^**^	.19	.52^**^				
16. Neg. act. emots. (aft)	−.17	−.35^*^	.02	.06	−.00^‡^	.26	.18	.15	−.22	−.48^**^	.76^**^	.78^**^	.27	−.24	−.56^**^			
17. Neg. deact. emots. (aft)	−.23	−.30	−.07	−.01	−.05	.28	.25	.06	−.28	−.50^*^	.72^**^	.87^**^	.19	−.28	−.55^**^	.84^**^		
18. Neutral emotion (aft)	.15	.03	−.27	.31	.14	−.19	−.28	.27	.51^**^	.16	.22	.08	.57^**^	.54^**^	.18	.13	.04	
19. Self-efficacy	.19	.14	.05	.24	−.00^‡^	−.09	−.14	.12	.39^*^	.50^**^	−.33	−.50^**^	.09	.53^**^	.55^**^	−.50^**^	−.50^**^	.08

Two-tailed test; *p < .05; **p < .01; pos. act. emot = positive activating emotions; pos. deact. emot = positive deactivating emotions; neg. act. emot = negative activating emotions; neg. deact. emot = negative deactivating emotions; bef. = before; dur. = during; aft = after; The values of -.00^†^and -.00^‡^ were −.002 and −.004, respectively.

Logistic regression analysis of performance was carried out to examine the relationship between assessed emotions and diagnostic accuracy in the BioWorld tasks, and the result of the Hosmer–Lemeshow test (*χ*^2^ (8) = 6.07, *p* = 0.64) indicated the logistics regression model’s goodness of fit. Binary logistic regression analysis for diagnostic accuracy revealed that PA emotions experienced before the task predicted good diagnostic accuracy (*β* = 2.01, *p* < 0.05) while ND emotions during the task predicted poor diagnostic accuracy (*β* = −1.61, *p* < 0.05) (Table 6). Multiple regression analysis on self-efficacy was also conducted to investigate the relationship between assessed emotions and students’ post task self-efficacy. The analysis revealed that PA and PD emotions significantly predicted self-efficacy (*F* (6,34) = 4.88, *p* < 0.01) and accounted for 37% of the variance (adjusted *R*^2^ = 0.37). In particular, PA emotions before the task (*β* = 0.41, *p* < 0.05) and PD emotions (*β* = 0.32, *p* < 0.05) were predictors of self-efficacy (Table 7).

**Table 6:** Logistic regression analysis of variables predicting diagnostic accuracy

Predictor	*B*	*ES B*	Odds Ratio
Before			
Positive activating emotions	2.01*	.92	7.48
Negative deactivating emotions	1.57	.98	4.81
Neutral emotions	−.57	.35	.57
During			
Positive activating emotions	−1.65	.86	.19
Negative deactivating emotions	−1.61*	.68	.20
Neutral emotions	.81	.46	2.24

Hosmer and Lemeshow test of goodness of fit, *χ*^2^ (8) = .64; *R*^2^ = .22 (Cox & Snell), .29 (Nagelkerke); *p < .05. **p < .01

**Table 7:** Simultaneous multiple regression analysis of variables predicting self-efficacy

Predictor	*B*	*SE B*	*β*
Before-task emotions			
Positive activating emotions	10.81	5.05	0.41*
Positive deactivating emotions	−5.65	3.50	−0.23
Negative deactivating emotions	7.21	6.99	0.16
During-task emotions			
Positive activating emotions	.63	4.84	0.02
Positive deactivating emotions	7.67	3.81	0.32**
Negative deactivating emotions	−11.84	4.31	−0.43

*R*^2^ = .46, *F* (6,34) = 4.88; *p < .05. **p < .01

## Discussion

The present study aimed to collect and examine validity evidence for the J-MES in terms of Kane’s four elements of validity arguments (Fig. 2; Kane, 2013). To the best of our knowledge, this is the first study to assess emotions in East Asian medical trainees using a scale based on control-value theory.

### Scoring and generalization inferences

We found that participants in the pilot study generally evaluated the J-MES high on clarity and usability, indicating that the TRAPD team translation model was effective. They also reported that the range of items adequately represented the emotions felt during the clinical diagnostic reasoning task. In addition, the quantitative response patterns measured by the J-MES were similar to those of the original study measured by the MES (Duffy et al., 2018), demonstrating the adequacy of the J-MES for measuring a wide range of emotions in Japanese medical students. However, students in the pilot study perceived that pride, surprise, and compassion on the J-MES did not translate well from the original MES, indicating that more validity evidence on these emotion-related items needs to be gathered the better to adapt the scale to Japanese culture.

### Extrapolation inference

We found that culture can influence the perception and manifestation of emotions, as clearly demonstrated in the responses to items pertaining to pride. The participants reported that although Japanese students feel pride, they rarely express it to others due to Japanese cultural values that discourage such feelings and expressions. Whether they express pride or not in a learning situation was found to depend on their relationship with their colleagues. These responses may be related to the Japanese cultural context where emotions are relationship-focused constructs. Mascolo et al., (2003) also report that Americans are encouraged to experience pride because doing so fosters the individual’s self-esteem and celebrates their achievement. In contrast, in the more collectivist East Asian culture, expressing pride is strongly discouraged because the individual’s achievements are not seen as due exclusively to the individual but as an outcome of one’s relationships with others who have aided the individual in achieving success. Imada and Ellsworth (2011) have shown that Americans tend to feel proud of themselves for successes and blame external factors for their failures whereas Japanese tend to feel lucky (i.e., external factors) for their successes and blame themselves for their failures. Students from more independent, western cultures credit themselves for their successes, and students from more interdependent, Asian cultures tend to attribute their successes to the situation or to others (Markus & Kitayama, 2010; Triandis, 2015). Therefore, for medical students in East Asia, it is unnatural to rate pride on an emotion scale since this behavior, when it is an expression of self-assertion, is generally disapproved in collectivist cultures (Furukawa et al., 2012). However, it is uncertain whether all Japanese medical students hold this view because more students in this era of globalism have prior exposure to Western culture. Some participants with international experience responded that they were able to understand the items pertaining to pride; thus, removing this item from the J-MES may be unjustified based on the cross-cultural differences observed in typical Japanese medical students who did not have international experience.

Our participants responded that the J-MES items pertaining to compassion were better suited to assessing learning environments where students interact with each other and with patients rather than in a software-based learning environment such as BioWorld where students work individually to collect and interpret data and attempt to make a diagnosis. Thus, it may be necessary to examine the suitability of compassion-related items on the J-MES for use in learning environments involving interactions with others, such as resuscitation simulations (Fraser et al., 2014), medical interview training (Nomura et al., 2017), or training in delivering bad news (Lajoie et al., 2014).

### Implication inference

We demonstrated that Cronbach's alpha of the J-MES was higher than the reference value of 0.6 for all three time points (i.e., before, during, and after the task), indicating adequate reliability. The correlation analyses showed that the emotions assessed by the J-MES were significantly correlated to the control-value theory constructs of perceived control, value, prior knowledge, and post-task self-efficacy. Regression analysis also showed that positive emotions positively predicted the students’ performance and/or self-efficacy while negative emotions negatively predicted their performance. These findings corroborated our prediction of the results based on previous studies using control-value theory and supported the utility of the J-MES by demonstrating a correlation between the assessed emotions and other variables of related constructs (Artino et al., 2010; Duffy et al., 2015, 2018; Lajoie et al., 2018).

Measured emotions are interesting and innovative variables in medical education because they serve both as predictors of medical learners’ and professionals’ performances as seen in the present study and as outcomes of their psychological states in learning and working environments. The future directions of this study will focus on using the J-MES as a monitoring tool for emotional states in medical learners and healthcare professionals in the real-world. For example, in didactic educational sessions, the J-MES may enables lecturers to change and adjust their lecture style based on the real-time, measured emotions of attendees. When healthcare professionals use the J-MES during their work-shift to monitor workers’ emotional wellness, the scale may be used to facilitate their emotional metacognition, facilitating decisions about when to rest or seek help during a shift or to support effective communication with colleagues in the workplace.

The small sample size in the present study may limit widespread uncritical application of our findings. While the internal validity was examined by assessing the J-MES's reliability, external validity (i.e., generalizability) was not thoroughly evaluated. Therefore, it remains unclear whether the J-MES can provide the same results with different learner training levels, medical schools, and educational environments. We conducted this study at a Japanese medical school where courses are taught in English, a very unusual situation in Japan. While the consequent small sample size and the potential selection bias may affect the generalizability of our findings, the unique characteristic of this university furnished the cross-cultural perspective needed to assess the validity of the J-MES and provided an initial window into emotion research in medical education in Japanese culture.

Further validation studies of the J-MES will be conducted to collect more validity evidence with a larger sample size to enable us to employ a more robust statistical model, such as confirmatory factor analysis or structural equation modeling. Further, the J-MES should be tested in other learning environments, such as classrooms and workplaces. Workplace-based assessment, such as milestones and entrustable professional activities (EPAs), has become mainstream in medical education assessment, and researchers and clinical teachers may be interested in exploring the relationship between the J-MES and milestone/EPA scores. Furthermore, the methodologies for measuring more objective emotional data, such as physiological and psychological parameters (e.g., pulse rate, electrodermal activity, pupil dilation, and facial expression recognition), could be developed to complement the subjective nature of the J-MES.

In conclusion, we developed the J-MES by translating the MES into Japanese using the TRAPD team translation model to ensure linguistic equivalence between the Japanese and English survey versions. Validity evidence for the J-MES was evaluated by using Kane’s four elements (scoring, generalization, extrapolation, and implication inference). We found that the evidence aligned with control-value theory and that the J-MES can be an effective measurement tool for evaluating Japanese medical students’ emotions but that there were cultural variations in Japanese medical students’ perceptions and expressions of the emotion of pride. Further research is needed to confirm its robustness and cross-cultural applicability.

## Supplementary Information

Below is the link to the electronic supplementary material.

Supplementary file1 (PDF 708 kb)
