# Supplementary material for: Japanese medical learners’ achievement emotions: Accounting for culture in translating Western medical educational theories and instruments into an asian context
Source: Adv Health Sci Educ Theory Pract. 2021 May 12;26(4):1255–76. doi: 10.1007/s10459-021-10048-9 (PMC8452569; doi:10.1007/s10459-021-10048-9)
Supplement: Supplementary file 1 — Supplementary file1 (PDF 708 kb) [file 10459_2021_10048_MOESM1_ESM.pdf]

Appendix 1: Medial Emotion Scale

Emotions **BEFORE** the activity: Using the scale below, indicate how you currently feel *keeping in mind the activity you are about to begin*. For each emotion, please indicate the strength of that emotion by selecting the number that best describes the intensity of your emotion.

	1<br>Not at all	2<br>Very little	3<br>Moderate	4<br>Strong	5<br>Very strong
Confused	○	○	○	○	○
Hopeful	○	○	○	○	○
Bored	○	○	○	○	○
Proud	○	○	○	○	○
Sad	○	○	○	○	○
Anxious	○	○	○	○	○
Happy	○	○	○	○	○
Frustrated	○	○	○	○	○
Hopeless	○	○	○	○	○
Enjoyment	○	○	○	○	○
Ashamed	○	○	○	○	○
Compassionate	○	○	○	○	○
Surprised	○	○	○	○	○
Curious	○	○	○	○	○
Afraid	○	○	○	○	○
Grateful	○	○	○	○	○
Disappointed	○	○	○	○	○
Relieved	○	○	○	○	○
Angry	○	○	○	○	○
Relax	○	○	○	○	○

Appendix 2: Emotions from the Medical Emotion Scale classified according to valence and activation

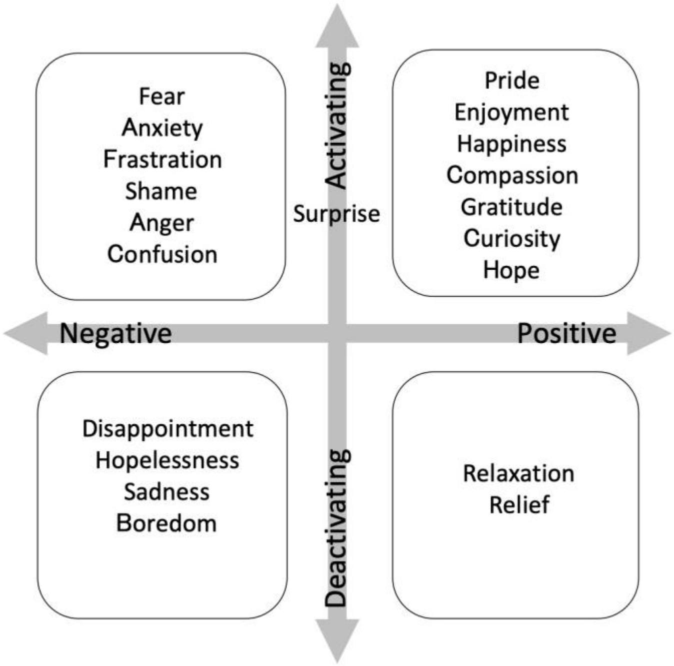

Appendix 3: Japanese version of Medial Emotion Scale

課題前の感情：今から実施される課題に対する、課題前の今の感情を、以下の尺度を用いて表現してください。それぞれの項目に、ご自身の気持ちを最も正確に示す番号を選んで書いてください。

	1<br>全く当てはまらない	2<br>ほとんど当てはまらない	3<br>ある程度当てはまる	4<br>ほぼ当てはまる	5<br>当てはまる
困惑している	○	○	○	○	○
期待している	○	○	○	○	○
うんざりしている	○	○	○	○	○
誇らしく思う	○	○	○	○	○
悲しい	○	○	○	○	○
不安だ	○	○	○	○	○
ウキウキしている	○	○	○	○	○
イライラしている	○	○	○	○	○
希望が持てない	○	○	○	○	○
楽しい	○	○	○	○	○
恥じている	○	○	○	○	○
思いやり	○	○	○	○	○
驚いている	○	○	○	○	○
好奇心	○	○	○	○	○
恐れている	○	○	○	○	○
感謝している	○	○	○	○	○
がっかりしている	○	○	○	○	○
安心している	○	○	○	○	○
怒っている	○	○	○	○	○
リラックスしている	○	○	○	○	○

Appendix 4: Interview guide and questions of pilot study (English & Japanese)

1. Opening sentence

I would now like to ask you your thoughts about the emotions that were presented on the questionnaire and how you interpreted and responded to them.

2. Questions

Did you feel that the emotions in the questionnaire captured the range of feelings that you experienced while doing the BioWorld?

- Show them copy of questionnaire for follow-up prompts
- Follow-up prompts:
  - ✓ Were there any emotions that you felt but were not listed on the questionnaire?
  - ✓ Were there any emotions that did not seem relevant?
  - ✓ Were there any that that seemed unclear or difficult to interpret?
  - ✓ Were the format, response options, ease of administration, and clarity of the instruction adequate?

1. 導入

このアンケートに記載されていた感情について、あなたがどのように解釈したり、回答するに至ったのかについて、これからお聞きしたいと思います。

2. 質問

アンケートで使用された感情を表わす表現は、ご自身が BioWorld で感じた気持ちをまんべんなく表現していたと思いますか？

- 追加の質問のためにアンケート用紙を示してください。
- 追加質問：
  - ✓ あなたが感じたけれども、アンケートにはなかった気持ちはありましたか、もしくはアンケートで使用された感情を表わす表現で、必要ないと思ったり、無関係な表現は何かありましたか？
  - ✓ わかりにくい、もしくは解釈するのが難しいと思った表現はありましたか？
  - ✓ アンケートに関して、内容やレイアウト、良いので分かりにくい、または解釈しにくいと感じたり、改善したほうが良いと思ったことはありますか？

Appendix 5: Coding scheme category of pilot qualitative analysis

Coding scheme	Elements
Suitability	How suitable are the questionnaire items? Which are unsuitable? Which items should be modified or re-ordered? What items should be added?
Clarity	Determines whether the individual questions are clear and whether the sore generally makes sense to the participant.
Usability	Appropriateness of the general format of the questionnaire; appropriateness of the response options contained in closed-ended questions; ease of administration; clarity of instruction
Additional emerging themes	Items requiring further investigation possibly due to cross-cultural issues

Appendix 6: Japanese version of the Academic Control Scale

	全く思わない	あまり思わない	どちらでもない	まあまあ思う	非常にそう思う
この授業で努力すれば、それだけ自分はさらによくなると思う。	1	2	3	4	5
大学においては、自分の結果は主として自分に責任があると思う。	1	2	3	4	5
この授業の成果は、自分自身で大いに制御可能だと思う。	1	2	3	4	5
もし、この授業の成績がよくなかったとしたら、それは自分の努力が足りなかったからだと思う。	1	2	3	4	5
大学で良い成績を取りたいなら，努力すればよいと思う	1	2	3	4	5

Appendix 7: Japanese version of the Motivated Strategies for Learning Questionnaire（Value subscale）

	全く思わない	あまり思わない	どちらでもない	まあまあ思う	非常にそう思う
もし適切な方法で勉強するならば、授業資料の内容を学ぶことができる。	1	2	3	4	5
授業での学習を他の授業で活用できると思う	1	2	3	4	5
もし授業資料を学習しないならばそれは自分の責任だ。	1	2	3	4	5
一生懸命勉強するならば、授業資料を理解できるだろう。	1	2	3	4	5
最も満足なことはできるかぎり授業内容を理解しようとすることだ。	1	2	3	4	5
授業資料は自分の学習に役立つと思う。	1	2	3	4	5
授業の内容が好きだ。	1	2	3	4	5

Appendix 8: Japanese version of post-task self-efficacy

次回、今回の学習活動を効率よく行うことや、似たような課題を上手にこなすことに対して以下の尺度を用いて、

どの程度自信があるか答えてください。

0	10	20	30	40	50	60	70	80	90	100
全く自信がない					ある程度自信がある					とても自信がある

Appendix 9: Screen capture of the performance comparison between Leaners and Expert in BioWorld

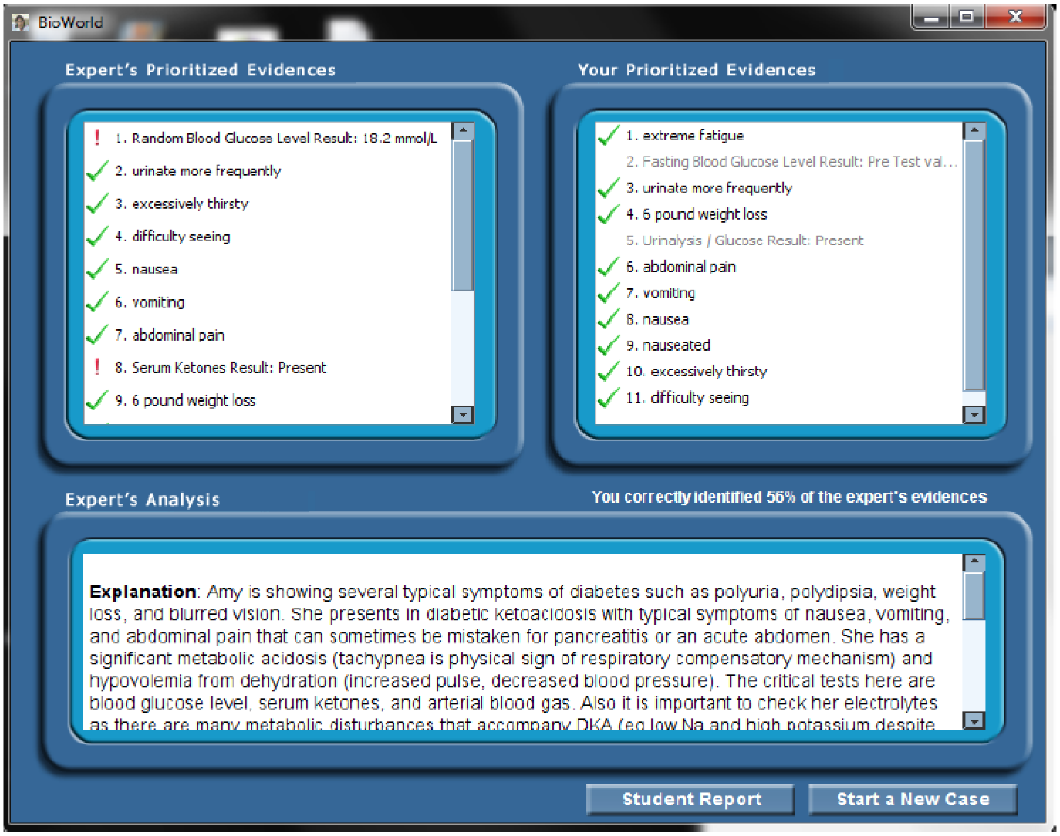

Note: red exclamation mark indicates non-match with expert; green checkmark indicates match with expert.

Appendix 10: Interview guide and questions of finalization phase study (English & Japanese)

1. Opening sentence

I would now like to ask you your thoughts about the emotions that were presented on the questionnaire and how you interpreted and responded to them.

2. Questions

Were there any emotions in the questionnaire that that seemed unclear or difficult to interpret?

- Show them copy of questionnaire for follow-up prompts
- Follow-up prompts:
  - ✓ Is the “pride” items was clear to understand?
  - ✓ Is the “compassion” item was clear to understand?
  - ✓ Is the “surprise” item was clear to understand?
  - ✓ Is surprise emotion positive or negative emotion for you?

1. 導入

このアンケートに記載されていた感情について、あなたがどのように解釈したり、回答するに至ったのかについて、これからお聞きしたいと思います。

2. 質問

✓ アンケートで使用された感情を表わす表現で、わかりにくいもしくは解釈するのが難しいと思ったものはありましたか？

- 追加の質問のためにアンケート用紙を示してください。
- 追加質問：
  - ✓ 「誇り」の項目は理解できましたか？
  - ✓ 「思いやり」の項目は理解できましたか？
  - ✓ 「驚き」の項目は理解できましたか？
  - ✓ 「驚き」という感情はあなたにとってポジティブな感情ですか？それともネガティブな感情ですか？

Appendix 11: Descriptive statistics for top five most intense discrete emotions over time in pilot study

	Top 5 positive emotions		Top 5 negative emotions	
	Discrete emotion	*M* (*SD*)	Discrete emotion	*M* (*SD*)
Time				
Before	Curious	3.8 (1.2)	Anxious	3.0 (1.4)
	Hopeful	2.8 (1.3)	Bored	2.2 (0.8)
	Happy	2.6 (0.8)	Confused	2.2 (1.2)
	Joyful	2.6 (1.2)	Hopeless	1.8 (1.2)
	Relaxed	2.6 (1.3)	Afraid	1.8 (1.2)
During	Curious	2.8 (0.7)	Confused	3.6 (1.0)
	Relaxed	2.6 (1.0)	Anxious	2.8 (1.7)
	Happy	2.4 (0.5)	Disappointed	2.8 (1.3)
	Proud	2.4 (0.5)	Ashamed	2.4 (1.3)
	Hopeful	2.0 (0.0)	Bored	2.2 (1.1)
After	Relaxed	3.4 (1.0)	Ashamed	2.6 (1.0)
	Curious	3.0 (0.6)	Sad	2.4 (1.4)
	Relieved	2.8 (1.5)	Anxious	2.4 (1.5)
	Happy	2.6 (1.0)	Disappointed	2.0 (1.1)
	Joyful	2.2 (1.2)	Afraid	2.0 (1.1)
